# Dynamic characteristics and fractal representations of crack propagation of rock with different fissures under multiple impact loadings

**DOI:** 10.1038/s41598-021-92277-x

**Published:** 2021-06-22

**Authors:** Bing Sun, Shun Liu, Sheng Zeng, Shanyong Wang, Shaoping Wang

**Affiliations:** 1 School of Civil Engineering, University of South China, Hengyang, 421001 Hunan China; 2 School of Resource & Environment and Safety Engineering, University of South China, Hengyang, 421001 Hunan China; 3 ARC Centre of Excellence for Geotechnical Science and Engineering, The University of Newcastle, Callaghan, NSW 2308 Australia; 4 College of Civil Engineering, Guangxi University, Nanning, 530004 China

**Keywords:** Engineering, Civil engineering, Petrology

## Abstract

To investigate the influence of the fissure morphology on the dynamic mechanical properties of the rock and the crack propagation, a drop hammer impact test device was used to conduct impact failure tests on sandstones with different fissure numbers and fissure dips, simultaneously recorded the crack growth after each impact. The box fractal dimension is used to quantitatively analyze the dynamic change in the sandstone cracks and a fractal model of crack growth over time is established based on fractal theory. The results demonstrate that under impact test conditions of the same mass and different heights, the energy absorbed by sandstone accounts for about 26.7% of the gravitational potential energy. But at the same height and different mass, the energy absorbed by the sandstone accounts for about 68.6% of the total energy. As the fissure dip increases and the number of fissures increases, the dynamic peak stress and dynamic elastic modulus of the fractured sandstone gradually decrease. The fractal dimensions of crack evolution tend to increase with time as a whole and assume as a parabolic. Except for one fissure, 60° and 90° specimens, with the extension of time, the increase rate of fractal dimension is decreasing correspondingly.

## Introduction

Fracture and instability of rock mass usually begin with original defects such as initial fissures, cracks and holes in rock mass. Especially under the disturbance of dynamic loads such as earthquake, blasting and excavation of underground engineering, the weak structural planes (e.g. faults and joints) in rock mass lose their initial equilibrium state. The further development and expansion of cracks lead to the decrease of rock strength, thus affecting the stability of rock mass. Therefore, the research on the dynamic properties and fracture propagation behavior of rock materials with different fissures has important engineering background and scientific significance. Research on dynamic response^1–5^ of rock materials under impact loading is very complicated. Loading rate^6–8^, material geometric characteristics^9^, energy dissipation^10–13^, and the non-linear problem of crack distribution^14,15^ are intertwined, which makes it difficult to analyze the stability of surrounding rock mass. Existing studies on rock mechanical properties and crack propagation are mostly concentrated in the low strain rate range. This kind of research has been systematically and perfectly studied by rock mechanics workers, and many achievements have been achieved^16–21^.

More and more geotechnical engineering such as mining, tunnel and deep ground, which makes the research on dynamic mechanical properties and crack propagation of rocks under medium and high strain rates become the frontier subject at present. In the study of dynamic mechanical properties, Wang et al.^22^ have studied the dynamic mechanical properties and failure modes of hard coal under different impact velocities by using SHPB. Haghnejad et al.^23^ have used three-dimensional discrete element program to study the effect of discontinuity of rock medium on the stability of mine slope under blasting load. Li et al.^24^ have carried out dynamic impact tests on prismatic marble specimens with a single fissure using an improved SHPB device. The effects of fissure with different dip and length on dynamic mechanical properties and fracture behavior have been analyzed. Yang et al.^25^ have analyzed the dynamic failure mechanism of rock at medium and low strain rates from three aspects of fracture morphology, energy absorption and mechanical parameters, and proposed a method for fast calculating dynamic compressive strength of rock using a single specimen. In the study of energy dissipation under dynamic loading, Li et al.^26^ have used SHPB test system to carry out dynamic splitting tensile test of rock specimens with single fissure, and have analyzed the energy dissipation law during the test process. Li et al.^27^ have conducted SHPB tests on rocks with two rough parallel joints, and have discussed the influence of the morphology of the two joint planes on the energy consumption of stress waves. In order to study the propagation behavior of cracks under dynamic loading, Jiang et al.^28^ have used gypsum three-dimensional printing materials to study the aggregation process of dynamic cracks. Wang et al.^22^ have measured the dynamic crack initiation and propagation toughness of rock materials by mixed experimental–numerical method. Wang et al.^29^ have studied the whole process behavior of crack propagation in an improved single cleavage seme-circle specimen (ISCSC) by combining the Split Hopkinson pressure bar (SHPB) experiment with numerical simulation.

In addition, in the process of crack propagation of rock materials, although the distribution of cracks is random and irregular, it has good self-similarity, i.e. fractal characteristics. Therefore, this kind of non-linear complex problem that is difficult to be quantitatively described can be solved by fractal theory. The application of fractal theory in rock crack propagation makes rock defects such as fissures and joints can be expressed by fractal dimension, which provides a theoretical basis for quantitative study of rock materials from micro-fracture to macro-failure process^30–38^. It has been found that the fractal dimension of crack distribution is usually related to stress^39–41^, geometric characteristics of rock specimens^42,43^, moisture content^44^ and fracture strength^45,46^. The size of fractal dimension mainly reflects the degree of flexure of cracks and the density of crack distribution. It can also be used to characterize the damage degree of rock materials^47^.

However, most of the above studies focus on the rock mechanics problems under the conditions of crack initiation, rock failure morphology, single mechanical parameters and single form of rock materials. There are few studies on the whole process behavior of crack propagation and rock mechanical properties of multi-fissure forms. Therefore, in this paper, the dynamic impact test of sandstone specimens with different fissure dip and number was carried out by using drop hammer impact test device. Based on the analysis of the dynamic mechanical properties of rock materials under different fissure forms and the evolution characteristics of the whole process of crack propagation, the energy transfer law between the test device and sandstone specimens during the dynamic impact process is proposed. At the same time, the failure process of sandstone is analyzed, and the fractal dimension of sandstone surface cracks after each loading is calculated by using fractal theory. The fractal growth model of sandstone surface cracks growth process is established, and the fitting function of fractal dimension of sandstone surface cracks in the process of sandstone instability and failure under cyclic loadings is obtained.

## Experiments

### Sample

**Figure 1:**
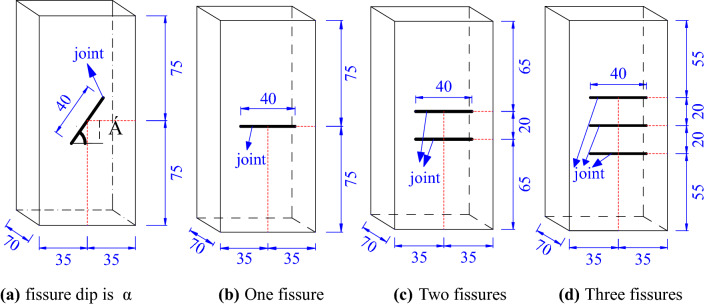
Model of prefabricated fissures (mm).

Yellow sandstone with a size of 70 mm × 70 mm × 150 mm was used in the experiment. The making process of fissure in specimen was as follows: Firstly, the specimen was punched with impact drill to obtain the diameter of the hole of 2 mm. Then the diamond wire was used to cut through the small hole along the direction of the crack. the width and length of the fissure were 1 and 40 mm respectively. There were three types of specimens: complete specimens, specimens with different fissure dips and different number of fissures. According to the different dips between fissure and loading direction, five kinds of specimens with dip of 0°, 30°, 45°, 60° and 90° were processed. According to the different number of fissures, four kinds of specimens with complete, one horizontal fissure, two horizontal fissures and three horizontal fissures were processed respectively. Three specimens were prepared for each type. Figure 1 is the distribution map of prefabricated fissures and α is the fissure dip. All specimens were polished to make the surface roughness less than 0.05 mm and the end surface perpendicular to its axis less than 0.25°.

### Experimental scheme

XJL-98 drop hammer impact testing machine is adopted for dynamic mechanical properties test. The maximum drop hammer height is 2 m, and the weight of heavy hammer is 1–5 kg. There are totally four groups: For group A, The weight of the heavy hammer is 4 kg; the falling height is 0.5 m, 1 m, 1.5 m and 2 m, and the specimens are sandstone with fissure dip of 45°. For Group B, The falling height is 2 m; the weight of the heavy hammer is 2 kg, 3 kg, 4 kg and 5 kg, and the specimens are sandstone with fissure dip of 45°. For Group C, The weight of the heavy hammer is 5 kg; the falling height is 2 m, and the rock specimens are with dips of 0°, 30°, 45°, 60° and 90° respectively. For Group D, the weight of heavy hammer is 5 kg; the falling height is 2 m, and the rock specimens are intact sandstone, sandstone with one fissure, two fissures and three fissures respectively. The detailed parameters of specimens are listed in Table 1.

**Table 1:** The detailed parameters of specimens.

Specimen	Height/m	Weight/kg	dip/°	Fissure number/strips	Specimen	Height/m	Weight/kg	dip /°	Fissure number/strips
A1	0.5	4	45	1	B1	2	2	45	1
A2	1				B2		3		
A3	1.5				B3		4		
A4	2				B4		5		
C1	2	5	0	1	D1	2	5	0	0
C2			30		D2				1
C3			45		D3				2
C4			60		D4				3
C5			90						

**Figure 2:**
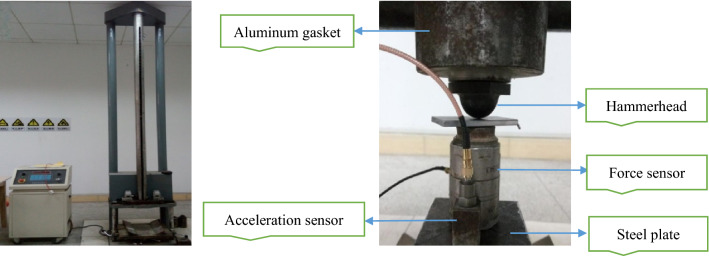
Experimental facility and the layout of the measuring device.

During loading, the hammer dropped freely and impacted the force sensor placed in the middle of the top of the sandstone sample. Then the sample was loaded by the force sensor, and the time history signal of the impact force was recorded. In the middle of the top of the sandstone sample, an acceleration sensor was arranged to measure the vertical acceleration of the sample. Vertical velocity and displacement can be obtained by integrating vertical acceleration. A strain gauge was attached to the compression area in the middle of the side of the sample to measure the strain time history signal of sandstone. All data were collected by dynamic signal analyzer. The experimental signal acquisition system consists of charge amplifier, force sensor, acceleration sensor, super dynamic strain gauge, dynamic signal acquisition instrument and computer. The layout of the measuring device is shown in Fig. 2.

## Results and discussion

### Energy conversion analysis

1. Momentum-impulse equilibrium relationship
  In drop hammer impact test, the gravitational potential energy was converted into kinetic energy when the hammer dropped. There are momentum-impulse equilibrium relations in the process as follows:
  1 $$\Delta \text{M}={\text{M}}_{0}-{\text{M}}_{\text{i}}=\text{I}$$
  In Eq. (1), ${\text{M}}_{0}=\text{m}\sqrt{2\text{g}\text{H}}$ is the initial momentum before contact with force sensor; ${\text{M}}_{\text{i}}$ is the initial momentum of the specimen after contacting with the force sensor when the hammer falls; $\text{I}$ is the impulse of the specimen, which can be obtained by integral of impact force–time history curve. In the impact process, the energy dissipated by friction during the falling of the heavy hammer is neglected. After the impact, the momentum of the heavy hammer and the aluminum gasket can be neglected, because the speed of the heavy hammer and the mass of the aluminum gasket on the force sensor is very small.
  Figure 3 shows the momentum-impulse equilibrium relationship under the experimental conditions, where the weight of the hammer is 4 kg and the falling height is 0.5 m, 1 m, 1.5 m and 2 m; the falling height is 2 m and the weight of the hammer is 2 kg, 3 kg, 4 kg and 5 kg. In a group of experiments where the weight of the hammer remains unchanged at 4 kg and the falling height changes, the momentum–impulse relationships coincide basically with the central line in the graph, which indicates that the momentum-impulse equilibrium relationships in this group are basically satisfied. For a group of experiments in which the falling height is 2 m and the hammer weight varies, the momentum-impulse relationships between the hammer weights of 2 kg and 4 kg also basically fall on the central line of the graph, which indicates that momentum and impulse are basically balanced under these two conditions. Although the impulse is greater than the momentum under the conditions of hammer weights are 3 kg and 5 kg respectively, after the test, it is found that this is due to the rebound of the hammer when it collides with the force sensor, which causes the momentum of the specimen slightly larger than it would have been without the rebound.

**Figure 3:**
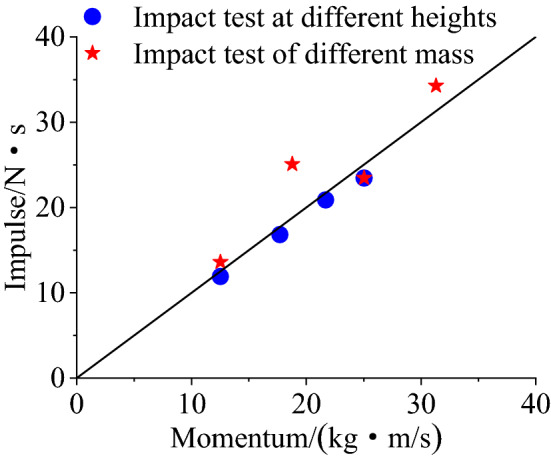
Momentum-Impulse equilibrium relationship.
2. Energy balance and conversion analysis
  In this impact test, the total energy of the system comes from the gravitational potential energy ${\text{E}}_{\text{p}}=\text{m}\text{g}\text{H}$ of the hammer, where $\text{m}$ and $\text{H}$ are weight and falling height of the hammer respectively. With the falling of hammer, gravity works on the hammer and gradually converts its gravitational potential energy into the kinetic energy of the hammer. Before the hammer collides with specimen, the gravitational potential energy of the hammer is converted into kinetic energy completely, and then works on the specimen by colliding with the specimen. During the collision between the hammer and the specimen, the total energy ${\text{E}}_{\text{p}}$ of the system is divided into two parts. One part of the energy is absorbed by the hammer, the aluminium gasket and the force sensor, and the other part is transformed into the impact force doing work on the specimen. The work ${\text{W}}_{\text{t}}$ of the impact force on the specimen can be obtained by integrating the impact force–displacement curve, that is ${\text{W}}_{\text{t}}=\int {\text{P}}_{\text{t}}\text{d}\text{S}$. Figure 4 shows the relationship between gravitational potential energy and work done by impact force, where the hammer weight is 4 kg, the falling height changes and the falling height remains unchanged, the hammer weight changes. From Fig. 4, for impact tests of the same mass and different heights, heavy hammer, aluminium gasket and force sensor consume most of the energy of the system, and the energy absorbed by sandstone samples only accounts for about 26.7% of the total energy. For impact tests of the same height and different mass, the energy consumed by heavy hammer, aluminium gasket and force sensor is relatively less, accounting for about 31.4% of the total energy, and the rest of the energy is entirely absorbed by sandstone samples.

**Figure 4:**
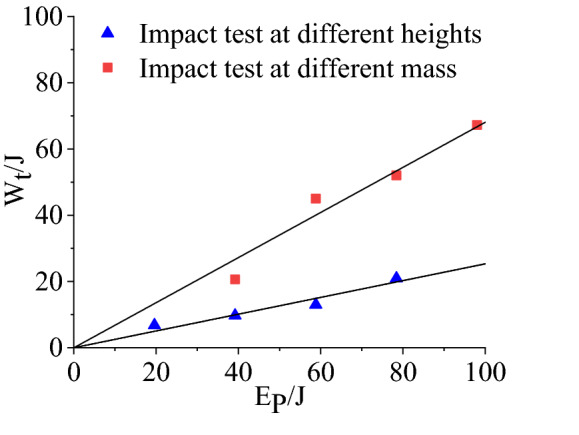
The relationship between gravitational potential energy and work done by impact force.

### Dynamic stress–strain relationship

**Figure 5:**
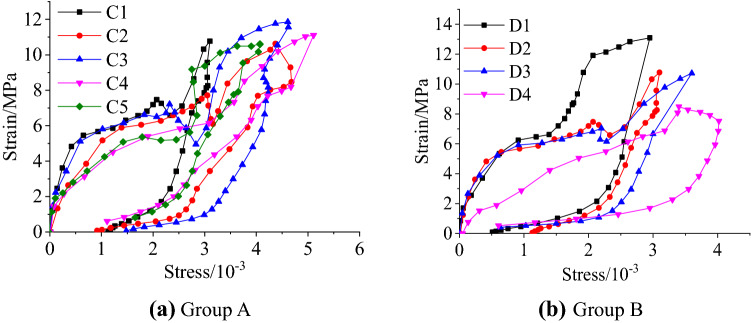
Dynamic stress–strain relationship.

The ultra-dynamic strain gauge records the stress-time signal and strain–time signal of the specimen. In order to study the failure mechanism of sandstone with different fissure dips and different fissure number under impact loading, according to the principle of drop hammer impact test, the stress-time signal and strain–time signal can be converted into the stress–strain signal of the specimen under impact compression.The following takes the first impact as an example to illustrate the stress–strain relationship of the specimen under impact loading. Similar to static loading, the dynamic compression deformation of the sandstone specimen has gone through the typical pore compaction stage, elastic stage, rapid crack development stage and descending section after fracture. Under dynamic impact, the compaction stage of the rock is extremely weak. It can be seen from Fig. 5 that only the stress–strain curve of the three-fissures specimen shows nonlinear changes in the initial stage, which is mainly due to the closure of the micro-fractures inside the rock under pressure. The compaction stage of the other samples is not obvious, and they directly enter the elastic deformation stage at the beginning of loading. Then enter the rapid development stage of the cracks, the stress–strain curve appears to a certain degree of depression, which is caused by the secondary collapse of the pores in the rock when the loaded stress exceeds the yield limit of most pores. After the elastic stage, a large number of pores collapse, and then arise a stress relaxation platform section. When loaded to the peak strength of the specimen, the specimen begins to fail, the failure process is flexible, and the stress drops slowly. The stress drop rate of the specimen depends on the integrity of the rock mass. When the sandstone contains more fissures, the internal micro-cracks are more likely to converge and nucleate, so it is easier to form penetrating cracks, resulting in the reduction of peak stress. After unloading, the specimen appears rebound deformation and unrecoverable residual deformation, which is mainly caused by the closing, slipping and dislocation of the structural surface during compression. 0°, 30°, 45°, single-fissure and double-fissures specimens all have pre-peak stress drop, which may be caused by localized tensile failure inside the specimen due to the heterogeneity of the rock.

### Dynamic mechanical parameters

The peak stress of the rock can reflect the ability of the rock to resist damage. There are many factors that affect the peak stress of the rock. On the one hand, it is the factor of the rock itself, and on the other hand, it is related to the relative size of the rock specimen, the processing condition and the loading rate. In order to study the ability of sandstone to resist damage under different fissure forms, the dynamic growth factor is used to reflect the change of the stress growth amplitude of the test block, that is, the dynamic growth factor formula is defined as follows:

2 $$\text{D}\text{C}\text{F}=\frac{{\sigma }_{d}}{{\sigma }_{s}}$$

where ${\sigma }_{d}$ (MPa) is the dynamic peak stress of the specimen; ${\sigma }_{s}$ (MPa) is the static peak stress of the specimen (the static peak stress of sandstone is 20 MPa); DCF is the dynamic growth factor of the specimen.

**Figure 6:**
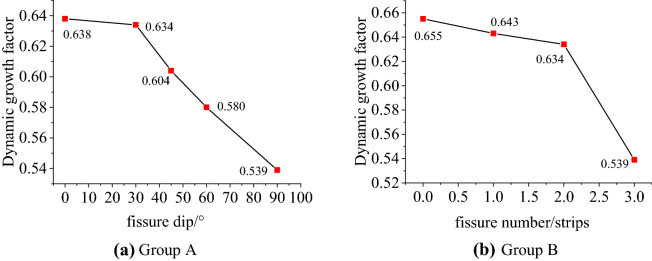
Relationship between dynamic growth factor and fissure dip and fissure number.

For samples with different fissure dips and different number of fissures, the dynamic growth factors under different working conditions are obtained, as shown in Table 2, and then plotted in Fig. 6.

**Table 2:** Dynamic growth factors under different working conditions.

Fissure dip/$^\circ$	DCF	Fissure number/strips	DCF
0$^\circ$	0.638	0	0.655
30$^\circ$	0.634	1	0.643
45$^\circ$	0.604	2	0.634
60$^\circ$	0.580	3	0.539
90$^\circ$	0.539

The analysis of Table 2 and Fig. 6 shows that the average dynamic peak stress of the sandstone specimen with the fissure dip of 0° is 12.76 MPa, and the average dynamic peak stress of the 30°, 45°, 60°, and 90° specimens is 12.67 MPa, 12.08 MPa, 11.59 MPa and 10.78 MPa, accounting for about 63.8%, 63.4%, 60.4%, 58%, 53.9% of the static peak stress of the complete specimen. The 90° specimen has the smallest dynamic peak stress, which is due to all the cracks of sandstone specimens developing from the top of the load along the 90° dip angle to the bottom. Therefore, the 90° specimen requires relatively less energy to fracture and its strength is relatively low. From the above analysis, it can be seen that the dynamic peak stress of the yellow sandstone specimens gradually decreases with the increase of the dip between the fissure and the loading section, and with the increase of the fissure dip, the peak stress decreases faster. In Fig. 6b, the average dynamic peak stress of the complete specimen is 13.1 MPa, and the average dynamic peak stresses of the single-fissure, double-fissures and three-fissures specimens are 12.86 MPa, 12.67 MPa and 10.78 MPa respectively, accounting for about 65.5%, 64.3%, 63.4% and 53.9% of the static peak stress of the complete specimen. It can be seen that the more the number of fissures in the rock specimen, the lower the strength, which is consistent with the theory.

**Figure 7:**
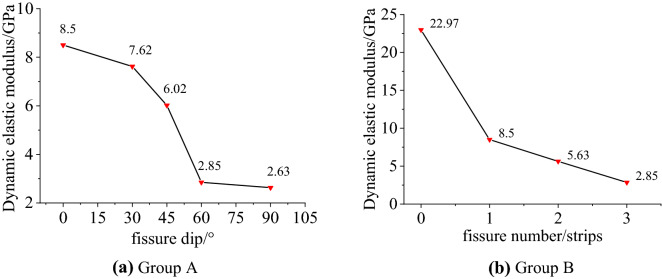
Relationship between dynamic elastic modulus and fissure dip and fissure number.

The dynamic elastic modulus is the tangent modulus of the stress–strain curve, that is, the slope of the straight or close to the middle of the stress–strain curve, which can be used to characterize the deformation properties of the rock. The decrease of the dynamic elastic modulus indicates that the strength of the rock is attenuating, and the degree of microcracks and damage is gradually increasing. Figure 7 shows the relationship between the dynamic elastic modulus and the fissure dip and the number of fissures. The dynamic elastic modulus decreases with the increase of the fissure dip and the number of fissures, indicating that with the increase of the fissure dip and the number of fissures, the more prone the sandstone sample is to produce microcracks and fracture behavior under the load. It can be seen from the figure that among the different fissure dips, the dynamic elastic modulus is greatly reduced with the 45° as the boundary, indicating that the fissure dip has a greater influence on the dynamic elastic modulus of the rock, and as the dip increases, the influence becomes greater. Compared with the dip of the fissures, the number of fissures has a greater impact on the dynamic elastic modulus of the rock. The dynamic elastic modulus of the specimen with a single fissure only accounts for 37% of the dynamic elastic modulus of the complete specimen. When there are a lot of micro-cracks, the dynamic mechanical properties of the rock will be greatly attenuated. In actual engineering, especially when there is dynamic disturbance, special attention should be paid to the impact of cracks on engineering safety.

### Crack dynamic propagation process

**Figure 8:**
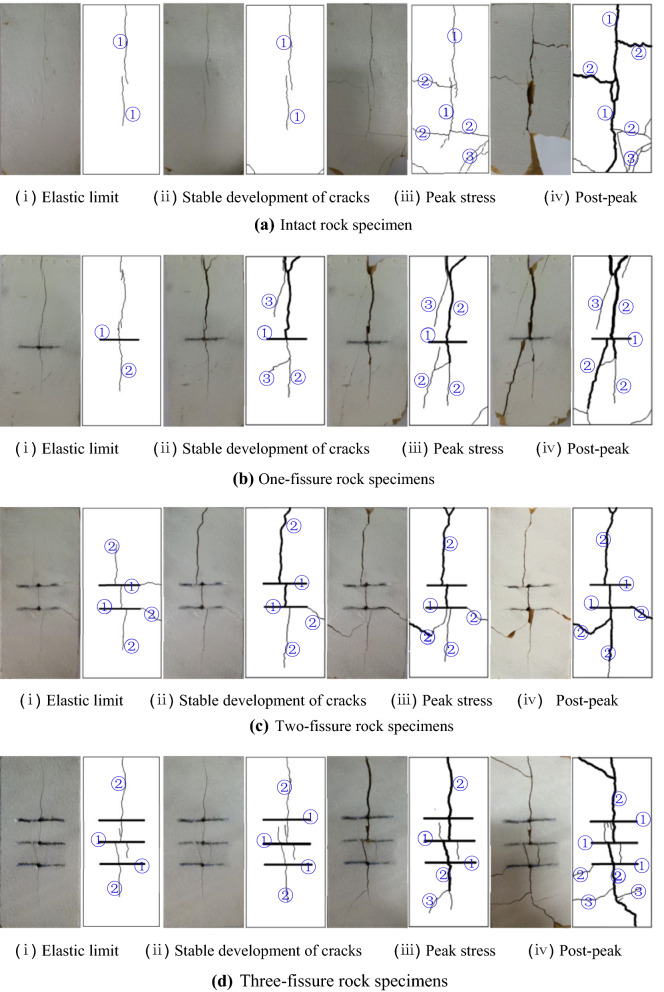

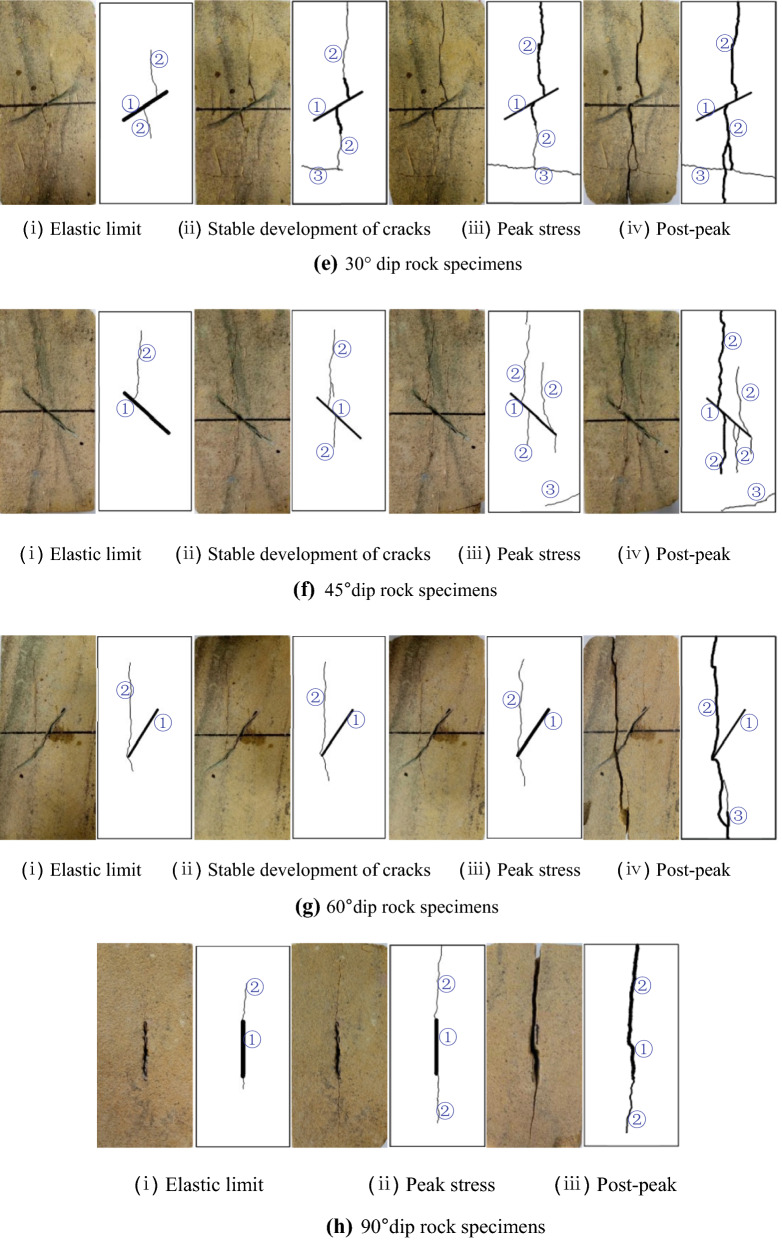
Crack growth process with different impact times (① represents main cracks, ② represents wing cracks, ③ represents secondary cracks).

Natural rock materials usually contain different forms of initial defects, which often change the mechanical properties and failure mechanism of rocks. When they are subjected to external loads of different degree, the internal defects of rock will continue to expand and evolve, which will lead to the deterioration of the bearing capacity of rock mass. Damage and fracture of rocks are the fundamental reasons of rock mass instability and various geological hazards. It is of great theoretical and engineering significance to study the damage and fracture process of rock materials under different fissure forms for predicting and evaluating the stability of engineering rock mass scientifically and accurately, and preventing the occurrence of major engineering geological hazards. Therefore, in order to study the effect of fissure morphology on the dynamic mechanical properties and crack propagation modes of rock materials, specimens with different fissure forms were processed by sandstone, and impact loading tests were carried out on drop hammer impact testing machine. In the tests, a hammer weight of 5 kg with a falling height of 2 m was selected, and the dynamic failure process of sandstone surface cracks during the test was recorded, as shown in Fig. 8.

Crack propagation process of specimens with different fissure numbers in Fig. 8 is summarized in Table 3. From Table 3, the longitudinal splitting cracks of intact specimens and one-fissure specimens start from the loading surface, with the initiation stress of 7.67 MPa and 7.32 MPa respectively. And the longitudinal splitting cracks of two-fissure specimens and three-fissure specimens start from the middle or near the middle of the prefabricated fissures, with the initiation stress of 2.6 MPa and 2.35 MPa respectively. It indicates that the dynamic compressive strength of the specimens with one fissure is lower than that of the complete specimens, but the reduction is small. The dynamic compressive strength of the specimens with two and three fissures is significantly lower than that of the complete specimen, and the dynamic compressive strength of the specimens with three fissures is smaller than that of the specimens with two fissures, but the difference is not significant. With the increase of impact loading, shear wing cracks appear at or near the tip of the splitting crack, following far-field cracks occur. Splitting cracks and shear cracks continue to expand and extend, and their widths also increase. When the stress–strain curve reaches the post-peak stage, energy concentrates around the cracks and gradually releases, resulting in internal crack failure and a large number of rock debris. Longitudinal splitting cracks penetrate along the loading direction, while (or later) transverse shear cracks penetrate perpendicularly to the loading direction. Then the specimen slightly distorts along the crack surface, which eventually leads to instability and failure. The crack initiation direction is parallel to the loading direction for both intact and jointed specimens, and the penetration failure of specimens is caused by tension-shear composite cracks. The more the number of prefabricated fissures is, the denser the cracks on the surface of the specimen are when failed, and the earlier the failure time is. From the above analysis, the crack propagation process is closely related to the number of prefabricated fissures. The more the number of prefabricated fissures is, the easier the initial cracks occur, the more dense the cracks are, and the easier the specimen is to destabilize and destroy.

**Table 3:** Crack propagation process of rock specimens with different prefabricated fissure numbers.

Fissure number/strips	Impact process	Stress (percentage of peak stress)/MPa	Loading time/ms	Crack propagation process description
0	1	7.67 (61.6%) Elastic limit	0.26	Starting from the loading end, longitudinal splitting cracks occur, and the cracks extend to the bottom
	2	8.94 (71.7%) Stable development of cracks	0.40	Cracks develop steadily and the width increases gradually, and shear cracks appear at the bottom of the specimen
	3	12.46 (100%) Peak stress	0.55	When the load reaches its peak value, the longitudinal splitting cracks continue to propagate along the loading direction, and begin to bifurcate, resulting in transverse cracks, and the bottom shear cracks gradually increase and grow
	4	7.63 (61.2%) Post-peak	0.80	Cracks continue to develop, the crack width increases, and the number is more dense, a large number of rock powder is produced, the bottom shear crack penetrates, and the specimen breaks from the shear crack
1	1	7.32 (61.9%) Elastic limit	0.25	Longitudinal splitting cracks begin at the loading end and continue to extend along the loading direction
	2	9.88 (83.5%) Stable development of cracks	0.42	The specimen has been destroyed at the loading point due to stress concentration. The cracks continue to propagate along the loading direction and begin to bifurcate, resulting in longitudinal shear cracks
	3	11.83 (100%) Peak stress	0.59	When the load reaches its peak value, the cracks continue to widen and lengthen, and the corner at the bottom of the specimen has been sheared
	4	7.59 (64.2%) Post-peak	0.74	The cracks become wider, longer and more dense, and the far-field cracks continue to initiate and propagate
2	1	2.60 (37.0%) Elastic limit	0.16	Longitudinal splitting cracks initiate and propagate from prefabricated fissures, and transverse wing cracks and shear cracks appear at the end of prefabricated fissures
	2	6.41 (91.2%) Stable development of cracks	0.36	Crack continue to propagate, the upper part has extended to the loading surface, and the lower part has continued to extend
	3	7.03 (100%) Peak stress	0.64	When the load reaches its peak value, the cracks continue to propagate and new shear cracks appear at the prefabricated fissures
	4	5.74 (81.7%) Post-peak	0.74	The crack continues to widen and lengthen, and the far-field crack continues to initiate
3	1	2.35 (40.0%) Elastic limit	0.14	Longitudinal splitting cracks are initiated at the loading end and prefabricated fissures of the specimens, and continue to propagate along the loading direction
	2	5.14 (87.6%) Stable development of cracks	0.18	Crack propagate steadily and continue to extend along the loading direction
	3	5.87 (100%) Peak stress	0.33	The cracks continue to widen and lengthen, and bifurcate
	4	3.34 (56.9%) Post-peak	0.53	Shear cracks at the loading end occur, bifurcation cracks appear at end the longitudinal cracks and the prefabricated fissures, and they propagate in the form of curve. Crack number is more dense, and a large number of rock powder is produced

In addition, crack propagation process of specimens with different fissure dips in Fig. 8 is summarized in Table 4. From Table 4, the longitudinal cracks of specimens with fissure dip of 30°–90° are initiated at a certain position at the tip or the middle part of prefabricated fissures, with the initiation stresses of 4.39 MPa, 7.61 MPa, 7.39 MPa and 5.51 MPa respectively. Theoretically, the initiation stresses of 30°–90° fissure specimens should be reduced in turn, while those of 30° specimens appear abnormal phenomenon in the test. It was found that the abnormal phenomenon may be related to the sampling position. With the increase of impact loading, shear cracks only appear on the surface of specimens with fissure dip of 30° and 45°, and longitudinal splitting cracks only occur on the surface of specimens with fissure dip of 60° and 90°. Whether splitting cracks or shear cracks, they are expanding and extending, and the crack width is also increasing. When the stress–strain curve reaches the post-peak stage, energy concentrates around the cracks and gradually releases, resulting in internal crack failure and a large number of rock debris. Longitudinal splitting cracks penetrate along the loading direction, while (or later) shear cracks penetrate staggeringly, resulting in instability and failure of the specimens. The penetration failure of specimens with fissure dip of 45°–90° is caused by longitudinal splitting cracks. The main penetration cracks of 30° fissure specimens have both tensile cracks and shear cracks. The failure time of 45° and 90° fissure specimens is the earliest. This is because the main failure mode of specimens under impact loading is splitting failure. The fissure dips of 45° and 90° make this kind of failure easier to occur, and the crack initiation direction is parallel to the loading direction. In summary, the crack propagation process is closely related to the prefabricated fissure dip. With the increase of the dip, the main crack gradually transits from splitting-shear crack to tension splitting crack.

**Table 4:** Crack propagation process of rock specimens with different prefabricated fissure dips.

Fissure dip/°	Impact process	Stress (percentage of peak stress)/MPa	Loading time/ms	Crack propagation process description
30	1	4.39 (47.8%) Elastic limit	0.18	Longitudinal splitting cracks occur from prefabricated fissures
	2	7.12 (77.6%) Stable development of cracks	0.28	Longitudinal cracks continue to propagate along the loading direction, the upper part has extended to the loading surface, the lower part has appeared transverse cracks, and the transverse cracks continue to propagate
	3	9.18 (100%) Peak stress	0.48	Load reaches its peak value, the crack widens gradually and the transverse crack extends to the side of the specimen
	4	5.57 (60.7%) Post-peak	0.72	Longitudinal cracks penetrate, cracks become wider and the number increases, a large number of rock powder appears
45	1	7.61 (62.5%) Elastic limit	0.19	A longitudinal splitting crack starts at the prefabricated fissure and extends upward
	2	11.65 (95.6%) Stable development of cracks	0.27	A new longitudinal splitting crack initiates from the prefabricated fissure and continues to propagate along the loading direction, while the original crack continues to propagate
	3	12.18 (100%) Peak stress	0.52	When the peak load is reached, many longitudinal cracks have appeared and are developing steadily. The bottom corner of the specimen has been sheared by shear cracks
	4	7.14 (58.6%) Post-peak	0.71	Cracks are becoming more and more dense, wider and longer
60	1	7.39 (57.3%) Elastic limit	0.19	A longitudinal splitting crack begins at the end of prefabricated fissures and continues to propagate along the loading direction
	2	11.27 (87.4%) Stable development of cracks	0.50	Longitudinal cracks continue to propagate and no new cracks are found
	3	12.90 (100%) Peak stress	0.61	Cracks continue to extend along the loading direction, and no new cracks are found
	4	9.11 (70.6%) Post-peak	0.72	Cracks penetrate and widen, and a new longitudinal short crack appears
90	1	5.51 (43.0%) Elastic limit	0.13	A longitudinal splitting crack initiates along the end of prefabricated fissures and continues to extend along the loading direction
	2	12.82 (100%) Peak stress	0.47	The original crack continues to extend along the loading direction, and no new crack is found
	3	7.88 (61.5%) Post-peak	0.58	The original longitudinal crack gradually widen and lengthen, and no new crack is found

### Fractal quantification of crack dynamic propagation

**Figure 9:**
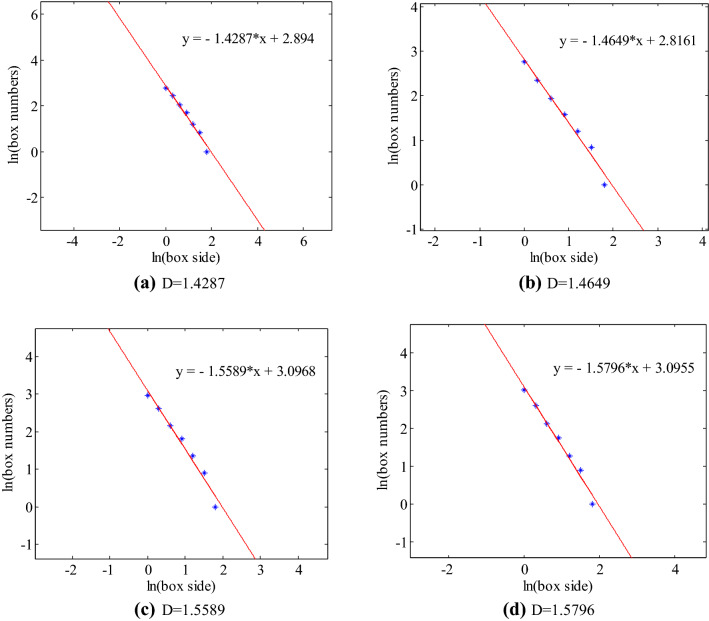
Intact rock sample box dimension fitting curve.

1. A method for calculating fractal dimension of crack distribution
  Fractal dimension is an important parameter for describing fractal, which can reflect the basic characteristics of fractal. Fractal characteristics of crack distribution can be obtained by fractal calculation of crack propagation and evolution process on sandstone surface. It is very helpful for further understanding the failure mechanism of rock under impact loading and putting forward reasonable precursor criterion of rock failure. With the different application of fractal, there are many definitions and calculation methods of fractal dimension. Similar dimension, Houston dimension, capacity dimension and box-counting dimension are common (Deng et al.^48^). This paper mainly calculates the fractal dimension of the surface crack image of the sample taken. Therefore, it mainly introduces the calculation method of the box-counting dimension of the two-dimensional digital image.
  Using the image processing and numerical calculation function of MATLAB, firstly, the image of surface crack of the sample is processed by gray level and binarization, and the related data is stored. Then the binary image is covered by a square box with the size of edge length r. The number of square boxes N(r) in the destroyed area of rock samples is counted, and the relevant data are saved. Among them, the relationship between the square edge length r and the number of square blocks N(r) is shown in Eq. (3) (Xie^49^),
  3 $${\log}N(\gamma )={\log}a-b{\log}\gamma$$
  where, both $a$ and $b$ are constants, and Eq. (4) can be obtained by taking logarithms on both sides of the Eq. (3),
  4 $$N\left(\gamma \right)= a{\gamma}^{-b}$$
  Fractal dimension D can be expressed as Eq. (5),
  5 $$D=-\underset{\gamma \to 0}{\mathrm{lim}}\frac{{\log}N\left(\gamma \right)}{{\log}\gamma }$$
2. Fractal quantification of crack propagation
  In this section, we mainly study the variation of fractal dimension of crack propagation process on sandstone surface under different fissure number and fissure dip. And the fractal growth model of crack growth process is intended to building-up. Firstly, the surface cracks of sandstone samples are segmented and extracted by using digital image processing technology and MATLAB software. Then, the program is written by MATLAB software to calculate the box dimension of the binary images after processing. Figure 9 shows an example of an intact sample. The box dimension of the surface crack of the intact sample is calculated by using MATLAB software. As shown in Fig. 9, the fitting degree of the curve is good, but when the box size is close to 2 (relatively large compared to other data points), the box numbers deviate a little from the fitting curve. It is proved that the selection of appropriate box size is very important in the calculation of box dimension. Among them, the opposite of the slope of the fitting curve is the box dimension. The box dimensions of surface cracks of specimens with different fissure number and different fissure dip are calculated in Tables 5 and 6 respectively.

**Table 5:** Fractal dimensions of surface cracks of specimens with different fissure number.

Fissure number/strips	Impact process	Fractal dimension	Fissure number/strips	Impact process	Fractal dimension
0	1	1.4287	1	1	1.4644
	2	1.4649		2	1.5274
	3	1.5589		3	1.6876
	4	1.5796		4	2.1876
2	1	1.4515	3	1	1.4734
	2	1.5148		2	1.4846
	3	1.5378		3	1.5427
	4	1.5510		4	1.5818

**Table 6:** Fractal dimensions of surface cracks of samples with different fissure dip.

Fissure dip/°	Impact process	Fractal dimension	Fissure dip/°	Impact process	Fractal dimension
30	1	1.4311	45	1	1.4068
	2	1.4669		2	1.4150
	3	1.4907		3	1.4871
	4	1.5088		4	1.5025
60	1	1.4048	90	1	1.4238
	2	1.4384		2	1.4897
	3	1.4993		3	1.5323
	4	1.5385		4	–
  From Tables 5, 6 and Fig. 9, the fractal growth model of crack propagation can be obtained for specimens with different fissure number and different fissure dip, as shown in Fig. 10. Under impact loading, the surface cracks of sandstone have good fractal characteristics, and the fractal dimensions of crack evolution tend to increase with time as a whole. The change of fractal dimension is closely related to energy. Except for one fissure, 60°and 90° specimens, with the extension of loading time, the increase rate of fractal dimension is decreasing, that is, the rate of damage degree caused by impact loading is decreasing. In the early stage of loading, the impact energy is larger, and the damage rock subjected is accordingly greater. With the extension of time, the energy is constantly attenuating, and the damage caused by impact loading is smaller. Beyond a certain time range, the damage of rock caused by impact loading gradually disappears. After the test, it is found that the fractal dimension increasing range of one fissure, 60°and 90° specimens is increasing over time, which is due to the failure time of these three kinds of specimens is relatively short, resulting in the damage degree of rock is becoming more and more serious. In summary, under impact loading, the damage of rock is more serious in the early stage than in the later stage, which indicates that the fractal dimension increasing range is more obvious in the later stage than in the earlier stage. Therefore, fractal dimension can be used as a parameter to indicate the damage degree of rock.

**Figure 10:**
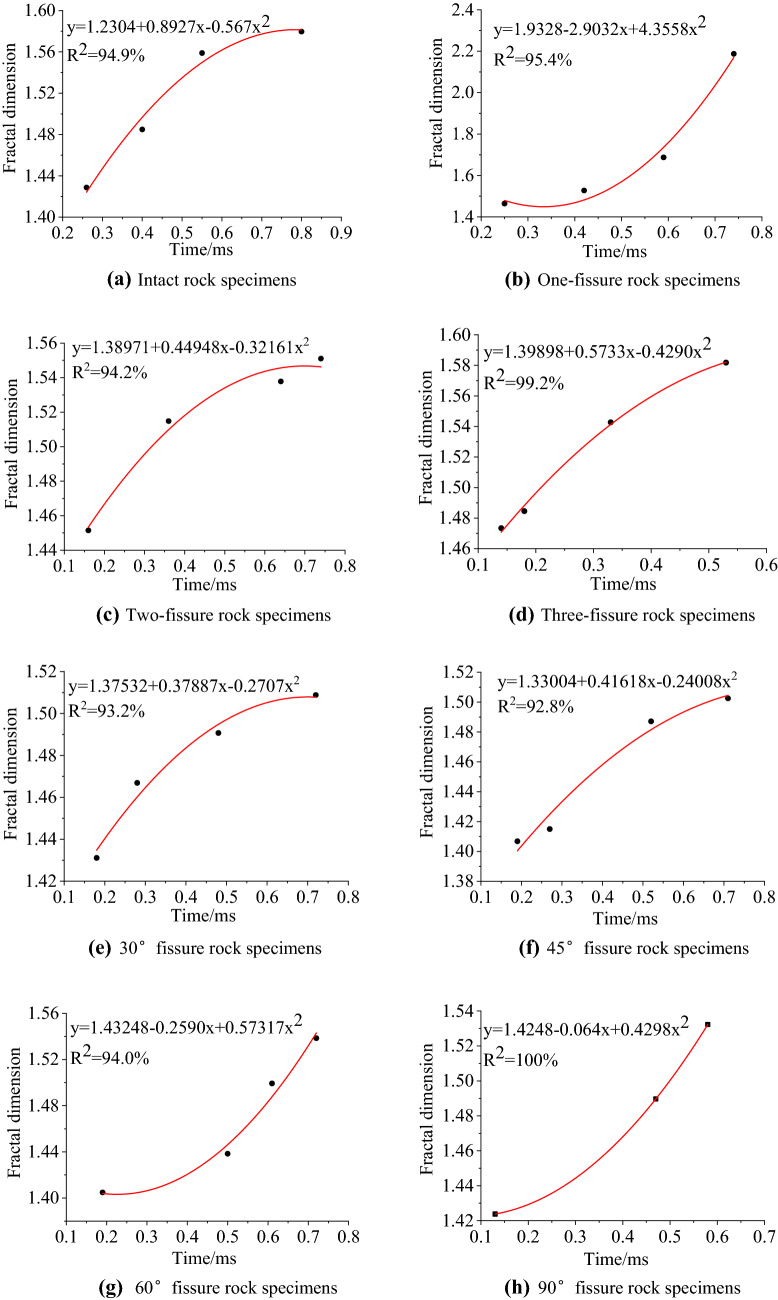
Fractal growth model of cracks.

## Conclusions

1. The impact process of the test satisfies the momentum-impulse conservation relationship. According to the energy conservation relationship, under the impact test conditions of the same mass and different heights, the energy absorbed by the sandstone sample only accounts for about 26.7% of the gravity potential energy of the weight; under the same height and different mass impact test conditions The energy absorbed by the sandstone sample accounts for about 68.6% of the total energy. The energy not absorbed by the sample is consumed by the weight, aluminum gasket and force sensor.
2. As the fissure dip increases and the number of fissures increases, the dynamic growth factor and dynamic elastic modulus of the fractured sandstone gradually decrease, and with a 45° dip as the boundary, the dynamic elastic modulus decreases significantly; the dynamic elastic modulus of the specimen with a single fissure only accounts for 37% of the dynamic elastic modulus of the complete specimen, which proves that the cracks have a great influence on the dynamic mechanical parameters of the rock.
3. Under the test conditions where the number of fissures is changed, the crack initiation direction is parallel to the loading direction, and the penetration failure of the specimen is caused by the tensile-shear compound crack. Under the test conditions where fissure dip changes, as the dip increases, the main crack gradually transitions from a split-shear crack to a tensile split crack.
4. In the fractal analysis of crack propagation, according to the idea of box dimension algorithm and the principle of digital image storage, an algorithm of box dimension of digital image based on MATLAB software is designed. The box dimension of rock surface crack under different test conditions is obtained, and the fractal growth model of crack with time is established. The fractal dimension can be used as a parameter to express the rock damage degree.

## Methods

### Momentum-impulse equilibrium relationship

In drop hammer impact test, the gravitational potential energy was converted into kinetic energy when the hammer dropped. There are momentum-impulse equilibrium relations in the process as follows:

1 $$\Delta \text{M}={\text{M}}_{0}-{\text{M}}_{\text{i}}=\text{I}$$

In Eq. (1), ${\text{M}}_{0}=\text{m}\sqrt{2\text{g}\text{H}}$ is the initial momentum before contact with force sensor; ${\text{M}}_{\text{i}}$ is the initial momentum of the specimen after contacting with the force sensor when the harmmer falls; $\text{I}$ is the impulse of the specimen, which can be obtained by integral of impact force–time history curve.

### The dynamic growth factor formula is defined as follows

2 $$\text{D}\text{C}\text{F}=\frac{{\sigma }_{d}}{{\sigma }_{s}}$$

where ${\sigma }_{d}$ (MPa) is the dynamic peak stress of the specimen; ${\sigma }_{s}$ (MPa) is the static peak stress of the specimen (the static peak stress of sandstone is 20 MPa); DCF is the dynamic growth factor of the specimen.

### A method for calculating fractal dimension of crack distribution

Fractal dimension is an important parameter for describing fractal, which can reflect the basic characteristics of fractal. Fractal characteristics of crack distribution can be obtained by fractal calculation of crack propagation and evolution process on sandstone surface. It is very helpful for further understanding the failure mechanism of rock under impact loading and putting forward reasonable precursor criterion of rock failure. With the different application of fractal, there are many definitions and calculation methods of fractal dimension. Similar dimension, Houston dimension, capacity dimension and box-counting dimension are common (Deng et al.^48^). This paper mainly calculates the fractal dimension of the surface crack image of the sample taken. Therefore, it mainly introduces the calculation method of the box-counting dimension of the two-dimensional digital image.

Using the image processing and numerical calculation function of MATLAB, firstly, the image of surface crack of the sample is processed by gray level and binarization, and the related data is stored. Then the binary image is covered by a square box with the size of edge length r. The number of square boxes N(r) in the destroyed area of rock samples is counted, and the relevant data are saved. Among them, the relationship between the square edge length r and the number of square blocks N(r) is shown in Eq. (3) (Xie^49^),

3 $${\log}N(\gamma )={\log}a-b{\log}\gamma$$

where, both $a$ and $b$ are constants, and Eq. (4) can be obtained by taking logarithms on both sides of the Eq. (3),

4 $$N\left(\gamma \right)= a{\gamma}^{-b}$$

Fractal dimension D can be expressed as Eq. (5),

5 $$D=-\underset{\gamma \to 0}{\text{lim}}\frac{{\log}N\left(\gamma \right)}{{\log}\gamma }$$
